# Randomized Prospective Double-Blind Studies to Evaluate the Cognitive Effects of Inositol-Stabilized Arginine Silicate in Healthy Physically Active Adults

**DOI:** 10.3390/nu8110736

**Published:** 2016-11-18

**Authors:** Douglas Kalman, Philip D. Harvey, Sara Perez Ojalvo, James Komorowski

**Affiliations:** 1 QPS-Miami Research Associates, 6141 Sunset Dr., Suite 301, South Miami, FL 33143, USA; 2 Miller School of Medicine, University of Miami, Miami, FL 33136, USA; pharvey@med.miami.edu; 3 Nutrition 21 LLC, JDS Therapeutics LLC, 1 Manhattanville Rd., Purchase, NY 10577, USA; sperezojalvo@nutrition21.com (S.P.O.); jkomorowski@nutrition21.com (J.K.)

**Keywords:** l-arginine, silicate, nitric oxide, cognitive function, mental flexibility

## Abstract

Inositol-stabilized arginine silicate (ASI; Nitrosigine^®^) has been validated to increase levels of arginine, silicon and nitric oxide production. To evaluate potential enhancement of mental focus and clarity, ASI (1500 mg/day) was tested in two double-blind placebo-controlled crossover (DBPC-X) studies using the Trail Making Test (TMT, Parts A and B). In the two studies, healthy males took ASI for 14 and 3 days, respectively. In the first study, after 14 days of dosing, TMT B time decreased significantly from baseline (28% improvement, *p* = 0.045). In the second study evaluating shorter-term effects, TMT B time decreased significantly compared to placebo (33% improvement, *p* = 0.024) in a 10-min period. After 3 days of dosing, TMT B time significantly decreased from baseline scores (35% improvement, *p* < 0.001). These findings show that ASI significantly improved the ability to perform complex cognitive tests requiring mental flexibility, processing speed and executive functioning.

## 1. Introduction

Inositol-stabilized arginine silicate (ASI; Nitrosigine^®^) is a novel sports nutrition ingredient previously shown to significantly enhance blood levels of arginine and silicon, while also increasing nitric oxide (NO) levels [1]. NO is synthesized endogenously from the recirculation of nitrites and from the nonessential amino acid l-arginine [2,3]. Although best known for its role in vasodilation leading to increased blood flow and cardiovascular health, NO has been shown to be involved in many physiological processes and to affect several organ systems [4]. There is evidence demonstrating the role of NO in exercise-induced vasodilation [5]. Supplementation of l-arginine has been shown to benefit hypertensive patients by correcting abnormal vasodilation. A meta-analysis of randomized, double-blind, placebo-controlled trials showed that oral l-arginine significantly lowered systolic and diastolic blood pressure compared with placebo [6]. In addition to demonstrated benefits in patients, arginine supplementation has been shown to enhance exercise performance in athletes and active adults through an increased time to exhaustion [7], improved recovery [8], and delayed muscular fatigue [6,9]. In healthy, minimally active males (19–33 years of age), ASI supplementation (1500 mg/day) for 4 days significantly increased pre-workout energy levels, increased muscle hyperemia, or measured size, over baseline measured by upper leg circumference post-exercise, and reduced biomarkers of muscle damage during recovery from exercise, supporting its role as a sports nutrition ingredient [10].

The evidence to support the benefits of l-arginine supplementation for cardiovascular disease [11] and exercise performance [12] are equivocal. It has been suggested that this may be explained by alterations in bioavailability or enzymatic breakdown. It has been demonstrated in rat models that ASI complex is more effective in increasing serum arginine levels than the commonly used arginine hydrochloride [13]. In a recent human, single-dose pharmacokinetic study, ASI supplementation increased blood levels of arginine between 30 min (18.2–24.1 μg/mL) and 5 h (20.3 μg/mL) after dosing, and increased blood levels of silicon for up to 1.5 h. Furthermore, blood levels of arginine and NO (salivary nitrite) were significantly elevated after 14 days of ASI supplementation [1]. In a recent study to evaluate the potential pharmacokinetic differences of ASI and Arginine Hydrochloride (ArgHCl) containing the same amount of arginine, a single-dose of ASI significantly increased plasma arginine levels at multiple time-points up to six hours post-dose, while ArgHCl supplementation did so for only one hour, suggesting that ASI is a more bioavailable source of arginine [14]. 

Silicon is an abundant trace element found in many plant based foods as well as many antacids and analgesic products. Silicon appears to enhance the bioavailability of l-arginine [13] and plays an important role in skin, hair, nails, immune and bone health [15,16]. Additionally, there is some evidence to suggest that silicon, especially as silicic acid, may protect vascular integrity during age-related vascular diseases [17]. There is also some evidence that silica (silicic acid) intake is associated with a reduced risk of Alzheimer’s disease and dementia, perhaps due to its ability to bind to and remove aluminum from the brain, suggesting a positive link between silica intake and cognitive function [18]. More research is warranted in this area.

Due to its role in blood circulation and potentially cerebral circulation, NO has been tested as a potential therapeutic strategy in treating and improving mild cognitive impairment [19,20]. The beneficial effects of NO in learning and memory are well documented, particularly in subjects with learning and memory impairment [21]. In a healthy population, supplementation of l-arginine has been shown to decrease stress and anxiety in healthy subjects [22]. It has been suggested that supplemental ASI may enhance exercise performance and mental focus and acuity through increased NO levels which results in increased blood flow and enhanced delivery of nutrients to the body and brain. Enhanced mental flexibility has been shown to benefit athletes when faced with quick decisions and associated adaptations often required during competition, especially in field sports and athletic events. In addition, elite athletes have been shown to score higher on tests assessing mental flexibility than non-elite athletes, therefore, enhanced mental flexibility may offer the “edge” that many athletes seek [23]. Research suggests that cognitive flexibility enhances performance by modulation of anxiety and stress during competition [24]. To further learn if supplementing with ASI in a physically healthy population can enhance mental focus and clarity, ASI (1500 mg/day) was tested in two double-blind placebo-controlled crossover (DBPC-X) studies using the Trail Making Test (TMT) as the cognitive outcomes measure. The TMT is a widely-used measure that is sensitive to a variety of different cognitive abilities and is very efficiently measured.

## 2. Materials and Methods

Any aspect of the work covered in the manuscript involving human patients has been conducted with ethical approval by Aspire IRB (Santee, CA, USA) on 6 November 2014, protocol numbers N21-ARM-2014 and N21-SORE-2014. Subjects were recruited by local advertisements in the newspaper, mailings and from the database in the greater Dade and Broward County, FL areas. Subjects underwent a phone screen prior to coming in for actual screening. In two separate clinical studies, healthy, non-smoking adult male subjects (only males were used to control for gender differences), aged 18–35 years, with Body Mass Index (BMI) 19–29.9 kg/m^2^, were enrolled after signing an informed consent. In DBPC-X Study #1, subjects (*n* = 11) with moderate exercise routines (defined as having performed weight training at least three times a week for at least the six months prior to starting the trial) took ASI or placebo daily for 14 days. Each subject was randomized to receive either double-blind ASI or placebo in a double-blind placebo controlled crossover design, with a seven-day washout period in between. Subjects were tested once at baseline (prior to ASI or placebo administration on Day 1), 10–15 min post-treatment on Day 1 and pre-treatment on day 14 in each period. They were instructed to refrain from exercise for 24 h prior to testing and to fast after midnight the night prior. In DBPC-X Study #2, subjects (*n* = 16) with limited exercise routines (defined as having exercised less than 150 min per week prior to participating in the study) were randomized to either ASI or placebo daily for 3 days, with a seven-day washout period followed by a crossover to the other treatment, in a double-blind randomized sequence. Subjects were tested once at baseline (prior to ASI or placebo administration on Day 1), 10–15 min post-treatment on Day 1 and pre-treatment on Day 3. 

The active treatment contained 1500 mg of ASI (arginine, silicon, inositol and potassium) and inactive ingredients—citric acid, maltodextrin, natural flavor, sucralose, acesulfame potassium, and FD&C Red 40—for flavor. The placebo treatment contained 0 g of ASI and inactive ingredients—citric acid, maltodextrin, natural flavor, sucralose, acesulfame potassium, and FD&C Red 40—for contents and flavor. The active and placebo products were given as single dose stick packs completely dissolved in 300 mL of water. 

Changes in cognition were measured using the Trail Making Test (TMT). The TMT is one of the most widely-used instruments in neuropsychological assessment as an indicator of cognitive processing speed and executive functioning [25]. The test consists of two parts (A and B). The dependent variable for each part is represented by the time to completion of the tasks. TMT A involves connecting an ascending sequence of 25 numbers. TMT B involves connecting an alternating sequence of 25 numbers and letters. Faster times in TMT B are associated with enhanced visual search, speed of processing, mental flexibility, and executive functions under performance demands [26,27]. In DBPC-X Study #1, changes in TMT A and TMT B were measured pre and post daily doses (approximately 10–15 min) in terms of changes from baseline on Day 1 and on Day 14. In DBPC-X Study #2, changes in TMT A and TMT B were measured pre and post daily doses in terms of changes from baseline on Day 1 and on Day 3.

Changes in safety parameters, including blood pressure, heart rate, adverse events and subjective remarks, were measured throughout both studies. 

A *p*-value of less than or equal to 0.05 was used to indicate statistical significance and values less than 0.10, but greater than 0.05 were deemed trending towards significance. The main efficacy analysis was conducted on a Per-Protocol basis with the main efficacy assessments involving tests of between-product endpoints and secondary assessments involving tests of within-product endpoints. All continuous variables were tested by the paired Student *t* test and analysis of covariance (ANCOVA) tests. All statistical analysis and graphs were completed using SPSS v. 22 (IBM Analytics, Armonk, NY, USA), R statistical/graphical programming system ver. 2.15.3 (The R Foundation, Redmond, WA, USA) and Microsoft Excel 2013 (The R Foundation).

## 3. Results

**Figure 1:**
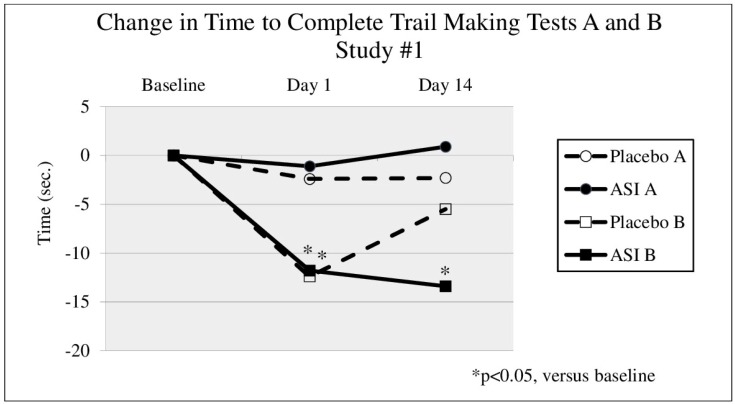
Change in time from baseline to complete Trail Making Tests (TMT) A and B, 10–15 min after a single dose and pre-dose after 14 days of inositol-stabilized arginine silicate (ASI) supplementation.

**Figure 2:**
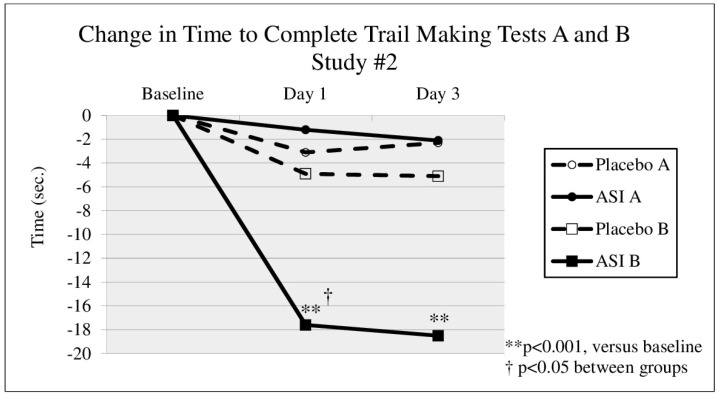
Change in time from baseline to complete Trail Making Tests (TMT) A and B, 10–15 min after a single dose and pre-dose after 3 days of inositol-stabilized arginine silicate (ASI) supplementation.

Subject demographics are presented in Table 1. In either study, no significant changes were seen in TMT A times between groups (Figure 1 and Figure 2). In the first study (DBPC-X, Study #1) TMT B (Figure 1) time significantly decreased after 14 days of treatment by 13.4 s in the ASI group (*p* = 0.045) from a baseline time of 47.3 s (an effect size of d = 0.45), compared to a non-significant decrease of 5.5 s in the placebo group (*p* = 0.067). In the second study (DBPC-X Study #2), approximately 10 min after taking the first dose, TMT B time (Figure 2) decreased by 17.6 s in the ASI group (*p* = 0.001) from a baseline time of 52.7 s (an effect size of d = 0.80), compared to a decrease of 4.9 s in the placebo group (*p* = 0.384). The change in TMT B time after 10 min was statistically significant between groups (*p* = 0.024). After 3 days of dosing, TMT B time (see Figure 2, day 3) decreased 18.5 s compared to baseline (*p* < 0.0005) (an effect size of d = 0.70), whereas the placebo group decreased 5.1 s (*p* = 0.517). No safety concerns were raised by these studies.

## 4. Discussion

It has been suggested that supplemental ASI may enhance exercise performance, mental focus and acuity through increased NO levels which results in increased blood flow and enhanced delivery of nutrients to the body and brain. Therefore, to confirm reports of enhanced focus and mental clarity, ASI (1500 mg/day) was tested in two double-blind, placebo-controlled crossover (DBPC-X) studies using TMT as the cognitive outcomes measure.

The study results indicate that complex, but not simple, processing speed tasks are enhanced by treatment with ASI. Evidence of sustained benefits, exceeding those of a practice effect, are seen in both studies, with changes from baseline to endpoint in Trail Making Test Part B that are quite consistent. While expected practice effects were seen in the placebo group, the reductions in completion times seen with ASI supplementation were greater than would be expected from simple practice effects alone. Reductions in completion times seen with ASI supplementation had effect sizes of d = 0.45, d = 0.70 and d = 0.80, which shows the strength of the effect to be considerable (from medium to large effect). The data also demonstrates benefits seen with acute supplementation, within 10 min of dosing, which may suggest a benefit immediately prior to competition or exercise. Performing TMT B requires executive functioning, speed and working memory. ASI has previously been shown to increase NO and it has been reported that NO plays a role in memory and that small changes in its local concentration are a key factor in determining its action [28]. It should be noted that no safety issues were seen with single and repeated use over 14 days.

Enhanced mental flexibility has been shown to benefit athletes when faced with quick decisions and associated adaptations often required during competition, especially in field sports such as football (American soccer) [23]. In addition, the ability to efficiently allocate attention is an important factor for success in all sports. The ability to multitask may help an athlete save energy through more efficient processing, thus also allowing them to perform better than before. Skilled athletes who adapt to rapid changes in visual information are able to allocate their attention more effectively than less skilled athletes [29]. They are then able to use visual scanning techniques as well as speed and anticipation to make changes in their performance [30]. This enhanced mental flexibility allows the athlete to adjust his or her “game” faster than their peers. Furthermore, a study of female collegiate lacrosse players demonstrated that TMT B scores were positively related to less lacrosse-shot error or less unsuccessful shots [31]. This suggests that successful sports performance encompasses a complex interaction of physical and cognitive skills [23] and that perhaps even a small improvement can make an impact on performance. Therefore, the improvements in complex processing speed with ASI supplementation reported in this study, as measured by TMT B, suggest benefit as a cognitive enhancing nutritional ingredient with specific applications in sports, competitions, and other athletic activities.

Cardiorespiratory fitness (volume of maximum oxygen consumption) has been positively associated with reasoning-related cognitive function [32]. Therefore, it is possible that we did not see an improvement in TMT A because the improvement was already seen as a function of fitness or because of potential ceiling effects in healthy populations varying in levels of physical activity. Similarly, Huijgen et al. (2015) found no difference in TMT A between elite and sub elite soccer players but did report that elite players had significantly better TMT B scores than sub-elite players. Therefore, future studies may want to consider adding a fully sedentary group [32].

Determining whether the improvements measured reflect improvements in executive functioning, as well as complex processing speed, will be important to clearly determine the effects of ASI supplementation on cognition. These studies require replication and expansion of the cognitive assessments. In addition, the benefits in cognitive functioning in athletes and cardiovascularly fit individuals warrants future studies. Future research will be needed to determine applications of these findings and to explore whether other cognitive domains are improved as well.

## 5. Conclusions

Results from two separate placebo-controlled clinical studies showed that daily doses of ASI significantly improved TMT B times, with effects seen in as little as 10 min after dosing and continued improvement with ongoing use. Expected practice effects were seen in placebo-treated subjects. Reductions in completion times seen with ASI supplementation were greater than would be expected from simple practice effects alone. Nitrosigine (ASI) led to a significant decrease in TMT B time compared to placebo, with an effect size of d = 0.80, which shows the strength of the effect to be considerable. Improvement in TMT B test times suggests improved complex processing speed in subjects treated with ASI. Improved mental flexibility is an area of potential athletic enhancement which is deserving of further research. 

## Figures and Tables

**Table 1:** Subject demographic data.

	DBPC-X Study #1	DBPC-X Study #2
Age	27.3 ± 4.2	25.5 ± 4.7 (17)
years	28 (21–34)	25 (19–33)
Gender		
Male	11 (100%)	16 (100%)
Total	11 (100%)	16 (100%)
Ethnicity		
Hispanic	10 (84%)	14 (82%)
Non-Hisp	2 (16%)	3 (18%)
Total	12 (100%)	17 (100%)
Race		
Black/AA	1 (8%)	5 (29%)
Caucasian	11 (92%)	12 (71%)
Total	12 (100%)	17 (100%)
Height	173.6 ± 8.3	175.8 ± 6.9
cm	174.5 (160–185.1)	175 (161–184.5)
Weight	77.7 ± 10.0	77.9 ± 12.6
kg	79.6 (55.6–90.1)	77.2 (58.8–99.2)
Body Mass Index (BMI)	25.7 ± 1.8	25.1 ± 3.1
kg/m^2^	25.5 (21.7–28.5)	25 (19.5–29.6)

## References

[1] Kalman D.S., Feldman S., Samson A., Krieger D.R. (2015). A clinical evaluation to determine the safety, pharmacokinetics, and pharmacodynamics of an inositol-stabilized arginine silicate dietary supplement in healthy adult males. Clin. Pharmacol..

[2] Parthasarathy D.K., Bryan N.S. (2012). Sodium nitrite the “cure” for nitric oxide insufficiency. Meat Sci..

[3] Zhao Y., Vanhoutte P.M., Leung S.W.S. (2015). Vascular nitric oxide: Beyond eNOS. J. Pharmacol. Sci..

[4] Clements W.T., Lee S.R., Bloomer R.J. (2014). Nitrate ingestion: A review of the health and physical performance effects. Nutrients.

[5] Maeda S., Miyauchi T., Kakiyama T., Sugawara J., Iemitsu M., Irukayama-Tomobe Y., Murakami H., Kumagai Y., Kuno S., Matsuda M. (2001). Effects of exercise training of 8 weeks and detraining on plasma levels of endothelium-derived factors, endothelin-1 and nitric oxide, in healthy young humans. Life Sci..

[6] Dong J.Y., Qin L.Q., Zhang Z., Zhao Y., Wang J., Arigoni F., Zhang W. (2011). Effect of oral l-arginine supplementation on blood pressure: A meta-analysis of randomized, double-blind, placebo-controlled trials. Am. Heart J..

[7] Yavuz H.U., Turnagol H., Demirel A.H. (2014). Pre exercise arginine supplementation increases times to exhaustion in elite male wrestlers. Biol. Sport.

[8] Rector T.S., Bank A.J., Mullen K.A., Tschumperlin L.K., Sih R., Pillai K., Kubo S.H. (1996). Randomized, double-blind, placebo controlled study of supplemental l-arginine in patients with heart failure. Circulation.

[9] Schaeffer A., Piquard F., Geny B., Doutreleau S., Lambert E., Mettauer B., Lonsdorfer J. (2002). l-arginine reduces exercise-induced increase in plasma lactate and ammonia. Int. J. Sports Med..

[10] Rood-Ojalvo S., Sandler D., Veledar E., Komorowski J. (2015). The benefits of inositol-stabilized arginine silicate as a workout ingredient. J. Int. Soc. Sports Nutr..

[11] Loscalzo J. (2004). L-Arginine and atherothrombosis. J. Nutr..

[12] Alvares T.S., Meirelles C.M., Bhambhani Y.N., Paschoalin V.M., Gomes P.S. (2011). l-arginine as a potential ergogenic aid in healthy subjects. Sports Med..

[13] Proctor S.D., Kelly S.E., Vine D.F., Russell J.C. (2007). Metabolic effects of a novel silicate inositol complex of the nitric oxide precursor arginine in the obese insulin-resistant JCR:LA-cp rat. Metabolism.

[14] Komorowski J., Perez Ojalvo S. (2016). A pharmacokinetic evaluation of the duration of effect of inositol-stabilized arginine silicate and arginine hydrochloride in healthy adult males. FASEB J..

[15] Jugdaohsingh R. (2007). Silicon and bone health. J. Nutr. Health Aging.

[16] Martin K.R. (2013). Silicon the health benefits of a metalloid. Met. Ions Life Sci..

[17] Buffoli B., Foglio E., Borsani E., Exley C., Rezzani R., Rodella L.F. (2013). Silicic acid in drinking water prevents age-related alterations in the endothelium-dependent vascular relaxation modulating eNOS and AQP1 expression in experimental mice: An immunohistochemical study. Acta Histochem..

[18] Rondeau V., Jacqmin-Gadda H., Commenges D., Helmer C., Dartigues J.F. (2009). Aluminum and silica in drinking water and the risk of Alzheimer’s disease or cognitive decline: Findings from 15-year follow-up of the PAQUID cohort. Am. J. Epidemiol..

[19] Austin S.A., Santhanam A.V., Katusic Z.S. (2010). Endothelial nitric oxide modulates expression and processing of amyloid precursor protein. Circ. Res..

[20] Talarowska M., Galecki P., Maes M., Orzechowska A., Chamielec M., Bartosz G., Kowalczyk E. (2012). Nitric oxide plasma concentration associated with cognitive impairment in patients with recurrent depressive disorder. Neurosci. Lett..

[21] Calabrese V., Mancuso C., Calvani M., Rizzitelli E., Butterfield D.A., Stella A.M. (2007). Nitric oxide in the central nervous system: Neuroprotection versus neurotoxicity. Nat. Rev. Neurosci..

[22] Smriga M., Ando T., Akutsu M., Furukawa Y., Miwa K., Morinaga Y. (2007). Oral treatment with l-lysine and L-arginine reduces anxiety and basal cortisol levels in healthy humans. Biomed. Res..

[23] Huijgen B.C.H., Leemhuis S., Kok N.M., Verburgh L., Oosterlaan J., Elferink-Gemser M.T., Visscher C. (2015). Cognitive functions in elite and sub-elite youth soccer players aged 13 to 17 years. PLoS ONE.

[24] Han D.H., Park H.W., Kee B.S., Na C., Na D.H.E., Zaichkowsky L. (2011). Performance enhancement with low stress and anxiety modulated by cognitive flexibility. Psychiatry Investig..

[25] Strauss E., Sherman E.M.S., Spreen O. (2006). A Compendium of Neuropsychological Tests: Administration, Norms, and Commentary.

[26] Kortte K.B., Horner M.D., Windham W.K. (2002). The Trail Making Test, Part B: Cognitive flexibility or ability to maintain set?. Appl. Neuropsychol..

[27] Reitan R., Wolfson D. (1997). The Halstead-Reitan Neuropsy-Chological Test Battery: Theory and Clinical Interpretation.

[28] Contestabile A., Monti B., Contestabile A., Ciani E. (2003). Brain nitric oxide and its dual role in neuroprotection/neurodegeneration: Understanding molecular mechanisms to devise drug approaches. Curr. Med. Chem..

[29] Enns J.T., Richards J.C. (1997). Visual attentional orienting in developing hockey players. J. Exp. Child Psychol..

[30] Sheppard J.M., Young W.B. (2006). Agility literature review: Classifications, training and testing. J. Sports Sci..

[31] Marsh D.W., Richard L.A., Verre A.B., Myers J. (2010). Relationships among Balance, Visual Search, and Lacrosse-Shot Accuracy. J. Strength Cond Res..

[32] Loprinzi P.D., Kane C.J. (2015). Physical exercise and its impact on psychology. Mayo Clin. Proc..

